# Immunogenicity and safety of an intramuscular split-virion quadrivalent inactivated influenza vaccine in individuals aged ≥ 6 months in India

**DOI:** 10.1080/21645515.2019.1565259

**Published:** 2019-03-12

**Authors:** Sharad Agarkhedkar, Jugesh Chhatwal, Rajeev Zachariah Kompithra, Sanjay K. Lalwani, Arun Narayan, Vinay Muninarayanaswam, Nithya Gogtay, Kristin Dotter, Viviane Gresset-Bourgeois

**Affiliations:** a Dr. D. Y. Patil Medical College, Hospital & Research Centre, Pune, Maharashtra, India; b Department of Pediatrics, Christian Medical College & Hospital, Ludhiana, Punjab, India; c Well Baby Immunization Clinic, Department of Pediatrics, Christian Medical College & Hospital, Vellore, Tamil Nadu, India; d Department of Pediatrics, Medical College Road, Pune, Maharashtra, India; e Department of Medicine, M.S. Ramaiah Medical College and Hospitals, Bangalore, India; f Department of Community Medicine, Mandya Institute of Medical Sciences, Mandya, Karnataka, India; g Department of Clinical Pharmacology, Seth GS Medical College & KEM Hospital, Mumbai, Maharashtra, India; h Medical Operations, Sanofi Pasteur, Swiftwater, PA, USA; i Global Medical Strategy, Sanofi Pasteur, Lyon, France

**Keywords:** Quadrivalent inactivated influenza vaccine, safety, immunogenicity, children, adults, India

## Abstract

A quadrivalent split-virion inactivated influenza vaccine (IIV4; Fluzone® Quadrivalent, Sanofi Pasteur) has been available in the US since 2013 for individuals aged ≥ 6 months. Here, we describe the results of an open-label, multicenter trial (WHO Universal Trial Number U1111-1143–8370) evaluating the immunogenicity and safety of IIV4 in Indian children aged 6–35 months and 3–8 years, adolescents aged 9–17 years, and adults aged ≥ 18 years (n = 100 per group). Post-vaccination hemagglutination inhibition titers for all strains in all age groups were ≥ 8 fold higher than at baseline (range, 8–51). At least 70% of participants in all age groups seroconverted or had a significant increase in titer for each strain. The most common solicited reactions were injection-site pain and tenderness, plus fever in participants 6–23 months and myalgia in older children and adolescents. All injection-site reactions and most systemic reactions were grade 1 or 2 and resolved within 3 days. Only three vaccine-related unsolicited adverse events were reported, all of which were grade 1 or 2 and transient. No immediate adverse events, adverse events leading to study discontinuation, adverse events of special interest, or serious adverse events were reported. This study showed that IIV4 was well tolerated and highly immunogenic in all age groups. This adds important data on the safety, tolerability, and immunogenicity of influenza vaccines in India.

Every year, influenza accounts for about 5%−10%^1,2^ of the approximately 43 million episodes of acute respiratory infection in India.^3^ During the peak rainy season, influenza represents 20%–42% of monthly acute medical illness hospitalizations.^1^ Since 2017, the government of India has recommended influenza vaccination for healthcare workers likely to be exposed to influenza virus, pregnant women, individuals at increased risk due to chronic illnesses, adults aged ≥ 65 years, and children aged 6 months–8 years.^4^ However, influenza control is complicated by genetic drift, varying seasonality in the different regions, and poor uptake of influenza vaccines.^1^

Trivalent influenza vaccines containing two A strains and a single B strain have been available in India since 2004.^1^ However, during the 1980s, influenza B split into two immunologically distinct lineages, Victoria and Yamagata, which now co-circulate.^5^ Because B-strain circulation varies between seasons and regions, differences between the vaccine and dominant circulating B lineage are common.^6,7^ Due to limited cross-lineage protection,^8-10^ quadrivalent influenza vaccines containing both B lineages are needed to reduce the risk of influenza illness and its associated morbidity and mortality.^11^ This is especially important in India, where the B strain of influenza appears to be particularly common.^12^

A quadrivalent split-virion inactivated influenza vaccine (IIV4; Fluzone® Quadrivalent, Sanofi Pasteur) has been available in the US since 2013 for individuals aged ≥ 6 months. Phase III clinical trials in adults aged ≥ 18 years^13,14^ and children aged 6 months–8 years^15^ demonstrated that IIV4 was as immunogenic as the comparator trivalent inactivated influenza vaccine for each of the three shared influenza strains and was more immunogenic for the additional B strain. In all clinical trials, IIV4 had a similar safety and reactogenicity profile as the comparator trivalent inactivated influenza vaccine (Fluzone®, Sanofi Pasteur).

To meet a request of the Indian health authorities, we performed an open-label, multicenter trial (WHO Universal Trial Number U1111-1143–8370) to evaluate the immunogenicity and safety of IIV4 in Indian children, adolescents, and adults. The study was conducted at eight sites between July 2015 and January 2017 and included 100 children aged 6–35 months, 100 children aged 3–8 years, 100 children and adolescents aged 9–17 years, and 100 adults aged ≥ 18 years. This was enough to provide 95% probability of observing adverse events with a true incidence of ≥ 3% in each age group. Participants aged 6–35 months could not have been primed (previously vaccinated with two doses of influenza vaccine approximately 28 days apart), and individuals aged ≥ 9 years could not have been vaccinated against influenza in the previous 9 months. Additional exclusion criteria and are listed in the Supplemental online information.

IIV4 was administered by intramuscular injection. Depending on the age group, participants received one or two vaccinations and either 7.5 or 15 µg of hemagglutinin per strain (see Table 1). Each age group was included and vaccinated separately, starting with the oldest and progressing to the next youngest. Prior to progression to the next youngest group, safety data collected up to 28 days after the first (or only) vaccination were reviewed and approved by the Drug Controller General of India.

**Table 1.** Study design, vaccines administered, and participant flow.

Vaccine characteristic or participant category	6–35 months	3–8 years	9–17 years	≥ 18 years
Vaccine formulation	NH 2016/2017	SH 2016	NH 2015/2016	SH 2015
Vaccine dose	2 × 0.25 ml (7.5 µg HA/strain) 28 days apart	2 × 0.5 ml (15 µg HA/strain) 28 days apart	1 × 0.5 ml (15 µg HA/strain)	1 × 0.5 ml (15 µg HA/strain)
Vaccine strains				
A(H1N1)	A/California/7/2009 X-179A	A/California/7/2009 X-179A	A/California/7/2009 X-179A	A/California/7/2009 X-179A
A(H3N2)	A/Hong Kong/4801/2014	A/Hong Kong/4801/2014	A/Switzerland/9,715,293/2013 NIB-88	A/South Australia/55/2014 IVR 175
B/Victoria	B/Brisbane/60/2008	B/Brisbane/60/2008	B/Brisbane/60/2008	B/Brisbane/60/2008
B/Yamagata	B/Phuket/3073/2013	B/Phuket/3073/2013	B/Phuket/3073/2013	B/Phuket/3073/2013
Participants				
Recruited	100	100	100	100
Voluntarily withdrew	1	1	0	0
Noncompliance with protocol	0	1	1	0
Completed the study	99	98	99	100

The immunogenicity and safety of vaccination with the quadrivalent split-virion trivalent inactivated influenza vaccine was assessed in an open-label trial conducted at eight sites in India between July 27, 2015 and January 27, 2017. Children 6 months to 8 years of age were recruited between August 3 and November 26, 2016. All other participants were recruited between July 27, 2015 and May 5, 2016. The trial was registered under WHO Universal Trial no. U1111-1143–8370. Participants aged < 9 years could not have been previous primed with an influenza vaccine and participants aged ≥ 9 years could not have been vaccinated against influenza within the previous 9 months. All participants or their legal representatives provided written, informed consent. Additional exclusion criteria and are listed in the Supplemental online information. Vaccines were formulated in phosphate-buffered saline, were thimerosal-, preservative-, and adjuvant-free, and were presented in prefilled syringes. Vaccines were administered by intramuscular injection into the thigh or deltoid muscle. The study was approved by the independent ethics committee for each site and was conducted in accordance with the Declaration of Helsinki and Good Clinical Practice. Abbreviations: NH, Northern Hemisphere, SH, Southern Hemisphere.

All adult participants completed the study. Four pediatric participants withdrew from the study for reasons other than an adverse event (Table 1). Of the participants who completed the study, more than half were male in the 6–35 month (54.5%) and ≥ 18 year (66.0%) age groups, whereas more than half were female (61.2%) in the 3–8 year age group. For the 9–17 years age group, nearly the same number of males and females were included.

HAI titers were measured from blood collected before vaccination (day 0) and 28 days after the last vaccination, as previously described.^16^ Pre-vaccination geometric mean hemagglutination inhibition (HAI) titers for each strain in each age group were mostly < 100 (1/dilution). Post-vaccination geometric mean HAI titers 28 days after the last vaccine dose (day 28 for children aged 6 months–9 years and day 56 for all others) were mostly > 1000 (Figure 1). Post-vaccination titers for all strains in all age groups were at least 8 fold higher than at baseline (range, 8–51), and at least 70% of participants in all age groups seroconverted or had a significant increase in HAI titer for each strain (range, 72%–96%). The lowest post-/pre-vaccination titer ratios and seroconversion rates were associated with high baseline titers, especially vs. A(H1N1) and A(H3N2) in participants aged 3–8 and 9–17 years. As found in other studies of inactivated influenza vaccines,^16-18^ post-vaccination titers decreased with age.

**Figure 1.**
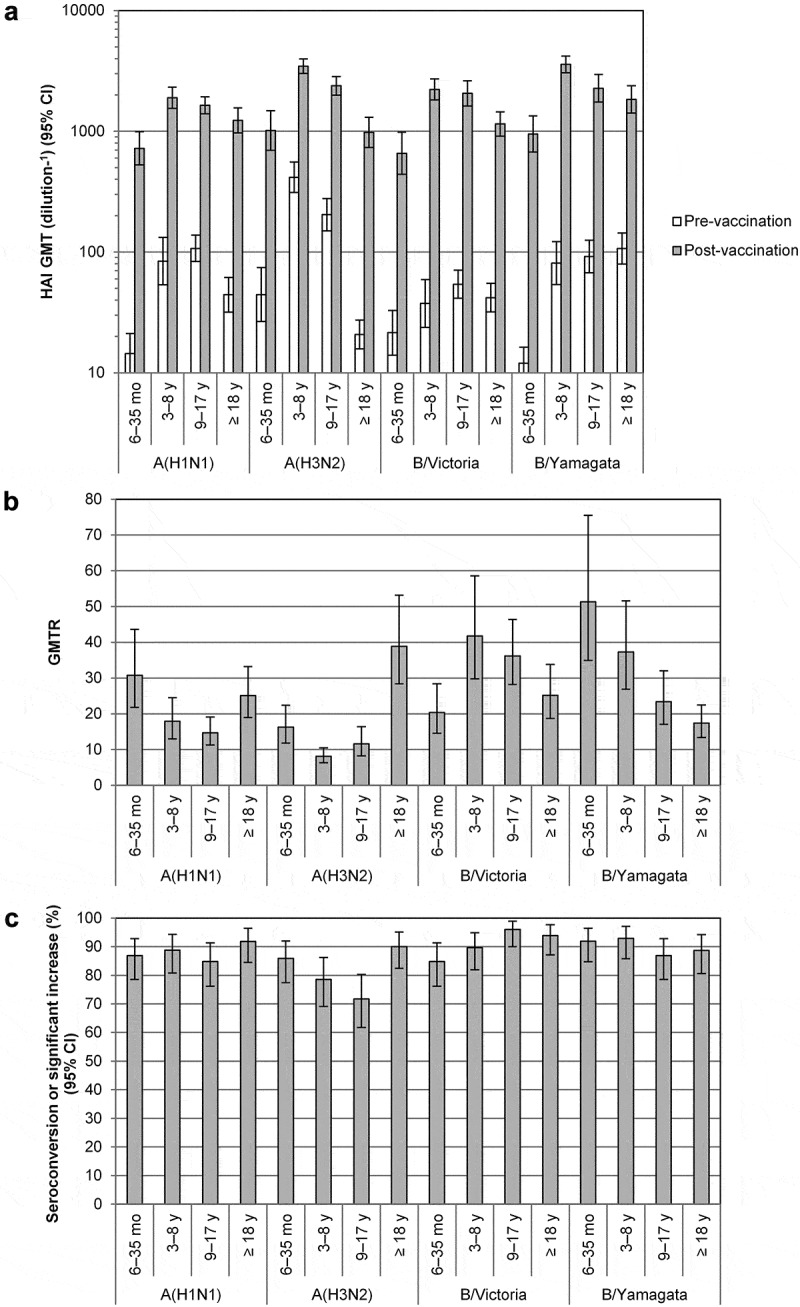
HAI Antibody response to vaccination with quadrivalent split-virion influenza vaccine. Blood was collected before vaccination (day 0) and 28 days after the last vaccination. Serum hemagglutination (HAI) titers were measured as described previously^16^ in all vaccinated subjects with data available and are expressed as 1/dilution. HAI titers under the lower limit of quantitation (10) were assigned a value of 5, and all HAI titers above the upper limit of quantitation (10,240) were assigned a value of 10,240. (A) HAI geometric mean titers (GMTs) and 95% confidence intervals (CIs) were determined from log_10_-transformed data using Student’s *t*-distribution with n−1 degrees of freedom, after which antilog transformations were applied to the results of calculations. (B) Geometric mean ratio of the individual post-/pre-vaccination HAI titer ratio (GMTR). (C) Proportion of participants seroconverting or with a significant increase in titer. Seroconversion was defined as a pre-vaccination HAI titer < 10 and a post-vaccination HAI titer ≥ 1:40. A significant increase was defined as a pre-vaccination HAI titer ≥ 10 and a ≥ 4-fold increase in HAI titer between pre- and post-vaccination. Statistical analysis was performed using SAS version 9.4 (SAS Institute, Cary, NC).

As reported by other investigators,^13-15^ the most common solicited reaction following vaccination with IIV4 was injection-site pain and tenderness, plus fever in participants aged 6–23 months and myalgia in older children and adolescents (Table 2). As also observed previously,^13^ frequencies of solicited reactions decreased with age. All solicited injection-site reactions and most solicited systemic reactions were grade 1 or 2, and most resolved within 3 days. The frequencies of solicited reactions observed here were lower than in other studies of IIV4,^13-15^ possibly due to lower reporting of reactions by parents in India, as found in other studies.^19,20^

**Table 2.** Solicited reactions.

	n (%)					
	6–23 months	24–35 months	3–8 years	9–17 years	18–64 years	≥ 65 years
Solicited reaction	(N = 71)	(N = 29)	(N = 100)	(N = 100)	(N = 70)	(N = 30)
*Any*	22 (31.0)	2 (6.9)	48 (48.0)	44 (44.0)	20 (28.6)	4 (13.3)
Grade 3	2 (2.8)	0 (0.0)	4 (4.0)	0 (0.0)	0 (0.0)	0 (0.0)
*Injection site*	11 (15.5)	2 (6.9)	41 (41.0)	36 (36.0)	20 (28.6)	4 (13.3)
Grade 3	0 (0.0)	0 (0.0)	0 (0.0)	0 (0.0)	0 (0.0)	0 (0.0)
Tenderness/pain	10 (14.1)	2 (6.9)	40(40.0)	35 (35.0)	20 (28.6)	4 (13.3)
Grade 3	0 (0.0)	0 (0.0)	0 (0.0)	0 (0.0)	0 (0.0)	0 (0.0)
Erythema	1(1.4)	0(0.0)	3(3.0)	4(4.0)	0(0.0)	0(0.0)
Grade 3	0 (0.0)	0 (0.0)	0 (0.0)	0 (0.0)	0 (0.0)	0 (0.0)
Swelling	2 (2.8)	0 (0.0)	3 (3.0)	4 (4.0)	0 (0.0)	0 (0.0)
Grade 3	0 (0.0)	0 (0.0)	0 (0.0)	0 (0.0)	0 (0.0)	0 (0.0)
*Systemic*	14(19.7)	1 (3.4)	17 (17.0)	21 (21.0)	3 (4.3)	1 (3.3)
Grade 3	2 (2.8)	0 (0,0)	4 (4.0)	0 (0.0)	0 (0.0)	0 (0.0)
Fever	9 (12.7)	0 (0.0)	8 (8.2)^a^	2 (2.0)^a^	0 (0.0)	0 (0.0)
Grade 3	0 (0.0)	0 (0.0)	2 (2.0)^a^	0 (0.0)^a^	0 (0.0)	0 (0.0)
Vomiting	2(2.8)	-	-	-	-	-
Grade 3	2 (2.8)	-	-	-	-	-
Abnormal crying	1(1.4)	-	-	-	-	-
Grade 3	0 (0.0)	-	-	-	-	-
Drowsiness	1(1.4)	-	-	-	-	-
Grade 3	0 (0.0)	-	-	-	-	-
Loss of appetite	3(4.2)	-	-	-	-	-
Grade 3	0 (0.0)	-	-	-	-	-
Irritability	5(7.0)	-	-	-	-	-
Grade 3	0 (0.0)	-	-	-	-	-
Headache	-	0 (0.0)	4 (4.0)	6 (6.0)	1 (1.4)	0(0.0)
Grade 3	-	0 (0.0)	1 (1.0)	0 (0.0)	0 (0.0)	0 (0.0)
Malaise	-	0 (0.0)	4 (4.0)	7 (7.0)	0 (0.0)	1 (3.3)
Grade 3	-	0 (0.0)	0 (0.0)	0 (0.0)	0 (0.0)	0 (0.0)
Myalgia	-	1(3.4)	9 (9.0)	16 (16.0)	2 (2.9)	1 (3.3)
Grade 3	-	0 (0.0)	0 (0.0)	0 (0.0)	0 (0.0)	0 (0.0)
Shivering	-	0 (0.0)	2 (2.0)	0 (0.0)	0 (0.0)	1 (3.3)
Grade 3	-	0 (0.0)	1 (1.0)	0 (0.0)	0 (0.0)	1 (1.0)

Solicited reactions were collected by participants or their legal representatives on diary cards for up to 7 days after each vaccination and analyzed in all participants vaccinated. Reactions were graded from 1 for the least severe to 3 for the most severe as describe in Table S1 in the Supplemental Online Information.

^a^N = 98

Only three vaccine-related unsolicited adverse events were reported, all of which were grade 1 or 2 and resolved within 2–6 days. These included gastroenteritis in a participant aged 6–23 months, upper respiratory tract infection in a participant aged 3–8 years, and rash in a participant aged 18–64 years. No immediate adverse events (i.e. occurring < 30 min after vaccination), adverse events leading to study discontinuation, or serious adverse events were reported. Also, no adverse events of special interest (Guillain-Barré syndrome, Bell’s palsy, encephalitis/myelitis, optic neuritis, Stevens-Johnson syndrome, toxic epidermal necrolysis, or febrile seizures) were reported. Thus, there were no new safety signals, and the vaccine appeared to be well tolerated.

Although children and adolescents were well represented, this study enrolled relatively few adults aged ≥ 65 years, which are a key target group for vaccination in India.^4^ Nonetheless, IIV4 has shown to be well tolerated and immunogenic in this age group in other countries.^13,14^ In addition, because the study covered more than one influenza season, a further limitation could be that not all participants received the same IIV4 formulation.

In conclusion, this study showed that IIV4 was well tolerated and was highly immunogenic in all age groups despite high baseline antibody levels in some cases and the different seasons and regions. This study adds important data on the safety, tolerability, and immunogenicity of influenza vaccines in India, which have been lacking, even though influenza vaccines have been in the Indian market since 2004.^1^ These results should help encourage switching to IIV4 or other quadrivalent vaccines to protect against influenza in India.
