# Supplementary material for: Immunogenicity and safety of an intramuscular split-virion quadrivalent inactivated influenza vaccine in individuals aged ≥ 6 months in India
Source: Hum Vaccin Immunother. 2019 Mar 12;15(4):973–7. doi: 10.1080/21645515.2019.1565259 (PMC6605869; doi:10.1080/21645515.2019.1565259)
Supplement: Supplemental Material [file khvi-15-04-1565259-s001.docx]

# Supplemental online information for “Immunogenicity and safety of an intramuscular split-virion quadrivalent inactivated influenza vaccine in individuals aged ≥ 6 months in India”

Additional exclusion criteria

- Receipt of any vaccine in the 4 weeks preceding the trial vaccination or planned receipt of any vaccine in the 4 weeks following trial vaccination
- Receipt of immune globulins, blood or blood-derived products in the past 3 months
- Known or suspected congenital or acquired immunodeficiency
- Receipt of immunosuppressive therapy, such as anti-cancer chemotherapy or radiation therapy, within the preceding 6 months
- Long-term systemic corticosteroid therapy (prednisone or equivalent for more than 2 consecutive weeks within the past 3 months)
- Self-reported history of seropositivity for human immunodeficiency virus, hepatitis B, or hepatitis C
- Systemic hypersensitivity to eggs, chicken proteins, neomycin, formaldehyde, octoxynol-9, or to any of the vaccine components, or history of a life-threatening reaction to the vaccine used in the study or to a vaccine containing any of the same substances
- Thrombocytopenia, contraindicating intramuscular vaccination
- Bleeding disorder, or receipt of anticoagulants in the 3 weeks preceding inclusion, contraindicating intramuscular vaccination
- For participants aged ≥ 9 years, current alcohol abuse or drug addiction
- Chronic illness that, in the opinion of the investigator, is at a stage where it might interfere with trial conduct or completion
- Moderate or severe acute illness/infection on the day of vaccination or febrile illness (temperature ≥ 38.0°C)
- Female aged ≥ 9 years pregnant, lactating, or of childbearing potential and not using an effective method of contraception

Solicited reactions

**Table S1. Solicited reactions and grading**

| **Reaction** | **Age group** | **Grade 1** | **Grade 2** | **Grade 3** |
| --- | --- | --- | --- | --- |
| Injection site |  |  |  |  |
| Tenderness | 6–23 months | Minor reaction when injection site is touched | Cries or protests when injection site is touched | Cries when injected limb is moved, or the movement of the injected limb is reduced |
| Pain | 2–11 years | Easily tolerated | Sufficiently discomforting to interfere with normal behavior or activities | Incapacitating, unable to perform usual activities |
|  | ≥ 12 years | No interference with activity | Some interference with activity | Significant; prevents daily activity |
| Redness, swelling | 6 months–11 years | > 0 to < 25 mm | ≥ 25 to < 50 mm | ≥ 50 mm |
|  | ≥ 12 years | ≥ 25 to ≤ 50 mm | ≥ 51 to ≤ 100 mm | > 100 mm |
| Systemic |  |  |  |  |
| Fever | 6–23 months | ≥ 38.0°C to ≤ 38.5°C | > 38.5°C to ≤ 39.5°C | > 39.5°C |
|  | ≥ 2 years | ≥ 38.0°C to ≤ 38.4°C | ≥ 38.5°C to ≤ 38.9°C | ≥ 39.0°C |
| Vomiting | 6–23 months | 1 episode per 24 h | 2–5 episodes per 24 h | ≥ 6 episodes per 24 h or requiring parenteral hydration |
| Abnormal crying | 6–23 months | < 1 h | 1–3 h | > 3 h |
| Drowsiness | 6–23 months | Sleepier than usual or less interested in surroundings | Not interested in surroundings or did not wake up for a feed / meal | Sleeping most of the time or difficult to wake up |
| Loss of appetite | 6–23 months | Eating less than normal | Missed 1 or 2 feeds/meals completely | Refuses ≥ 3 feeds/meals or refuses most feeds/meals |
| Irritability | 6–23 months | Easily consolable | Requiring increased attention | Inconsolable |
| Headache, malaise, myalgia, shivering | ≥ 2 years | No interference with activity | Some interference with activity | Significant; prevents daily activity |
